# 

**Published:** 1886-09

**Authors:** 


### CONTENTS—SEPTEMBER, 1886.

ORIGINAL COMMUNICATIONS—Gunshot Fractures of the Spinal Column, with Report of Three Cases. By Dr. W. J. Burt, Austin .......... 91
Forceps in Labor. By C. L. Gwyn, M. D., Galveston.. 106
A Method of Rendering Iodoform Inodorous. By C. K. Gregg, M. D., Mexico .......... 109
HOSPITAL PRACTICE—Malarial Paraplegia Relieved by Quinine. By Dr. C. H. Wilkinson, Galveston .......... 110
CORRESPONDENCE—The Jefferson Medical College and the International Medical Congress—Letter from Dr. Roberts Bartholow, Dean .......... 112
EDITORIAL—A "Notable Address" Indeed .......... 115
" The Good of the Cause .......... 115
" Dr. Bartholow's Position .......... 120
" A Dangerous Operation .......... 121
SOCIETY NOTES—Texas State Medical Association—Committee on Collective Investigation of Disease—Circular from the Chairman, Dr. S. R. Burroughs .......... 151
Texas State Medical Association—Dr. F. E. Daniel's appointment as Secretary .......... 123
Travis County Medical Association—Officers elected .... 123
Physicians' Mutual Benefit Association—Second death benefit—paid to Mrs. Sayres .......... 123
EDITORIAL NOTES—Too numerous to enumerate in half page 125–9
ADVERTISERS' NOTICES .......... 129

